# Protection of eIF2B from inhibitory phosphorylated eIF2: A viral strategy to maintain mRNA translation during the PKR-triggered integrated stress response

**DOI:** 10.1016/j.jbc.2023.105287

**Published:** 2023-09-22

**Authors:** Takuhiro Ito, Jennifer Deborah Wuerth, Friedemann Weber

**Affiliations:** 1 Laboratory for Translation Structural Biology, RIKEN Center for Biosystems Dynamics Research, Yokohama, Japan; 2 Institute of Innate Immunity, Medical Faculty, University of Bonn, Bonn, Germany; 3 Institute for Virology, FB10-Veterinary Medicine, Justus-Liebig University, Giessen, Germany

**Keywords:** integrated stress response, eIF2, eIF2B productive state, phosphorylated eIF2, eIF2B nonproductive state, PKR antagonist, coronavirus AcP10, picornavirus AiVL, phlebovirus NSs, Sandfly fever Sicilian phlebovirus, NSs mechanism, NSs structure

## Abstract

The integrated stress response (ISR) protects cells from a variety of insults. Once elicited (*e.g.*, by virus infections), it eventually leads to the block of mRNA translation. Central to the ISR are the interactions between translation initiation factors eIF2 and eIF2B. Under normal conditions, eIF2 drives the initiation of protein synthesis through hydrolysis of GTP, which becomes replenished by the guanine nucleotide exchange factor eIF2B. The antiviral branch of the ISR is activated by the RNA-activated kinase PKR which phosphorylates eIF2, thereby converting it into an eIF2B inhibitor. Here, we describe the recently solved structures of eIF2B in complex with eIF2 and a novel escape strategy used by viruses. While unphosphorylated eIF2 interacts with eIF2B in its “productive” conformation, phosphorylated eIF2 [eIF2(αP)] engages a different binding cavity on eIF2B and forces it into the “nonproductive” conformation that prohibits guanine nucleotide exchange factor activity. It is well established that viruses express so-called PKR antagonists that interfere with double-strand RNA, PKR itself, or eIF2. However recently, three taxonomically unrelated viruses were reported to encode antagonists targeting eIF2B instead. For one antagonist, the S segment nonstructural protein of Sandfly fever Sicilian virus, atomic structures showed that it occupies the eIF2(αP)-binding cavity on eIF2B without imposing a switch to the nonproductive conformation. S segment nonstructural protein thus antagonizes the activity of PKR by protecting eIF2B from inhibition by eIF2(αP). As the ISR and specifically eIF2B are central to neuroprotection and a wide range of genetic and age-related diseases, these developments may open new possibilities for treatments.

Protein synthesis is a central, energy-intensive process. Therefore, cells that become stressed by a shortage of molecular building blocks, accumulation of misfolded proteins, or viral infection, react by suppressing their mRNA translation. This comparatively radical and basic measure helps to prevent further damage, conserves energy for repair, and hinders the replication of viral intruders. In our review, we will on one hand focus on recent discoveries on the regulation of protein synthesis during a process known as the integrated stress response (ISR), and on the other hand describe a novel mechanism by which viruses are bypassing it. For this, we will first give a brief overview over the ISR, then describe the mechanism of eukaryotic translation initiation factor 2B (eIF2B) as the central hub of the ISR, summarize the antiviral action of the ISR kinase (protein kinase RNA-activated [PKR]), and eventually compare the well-established anti-PKR strategies of viruses with a novel mechanism targeting eIF2B. A short outlook on the translational potential of ISR inhibitors will also be provided.

As the regulation of eIF2B by cellular factors and viruses is a key aspect of health, infection, and disease, we are attempting to provide an update on these subjects that may be useful for cellular and structural biologists as well as for virologists.

## The integrated stress response

The ISR is initiated by different protein kinases that are responsive to specific types of stress. There are four well-established kinases (general amino acid control nonderepressible 2 [GCN2], heme-regulated inhibitor [HRI], protein kinase RNA-like endoplasmic reticulum kinase [PERK], and PKR) that trigger a stress response, and they all act at the rate-limiting step of translation initiation (1). GCN2 is activated by amino acid deprivation and UV exposure, HRI by protein misfolding in the cytosol (2, 3), PERK by protein misfolding in the endoplasmic reticulum (“ER stress”), and PKR by viral infection. In addition, a fifth ISR kinase, microtubule affinity-regulating kinase 2 (MARK2), was recently discovered to also react to proteotoxic stress (4), and microglia contain an ISR kinase, FAM69C (family with sequence similarity 69 member C), that responds to oxidative stress (5). The ISR kinases (extensively reviewed recently (1)), aside from the antiviral PKR, are not the subject of our article and will therefore not be discussed in detail.

**Figure 1:**
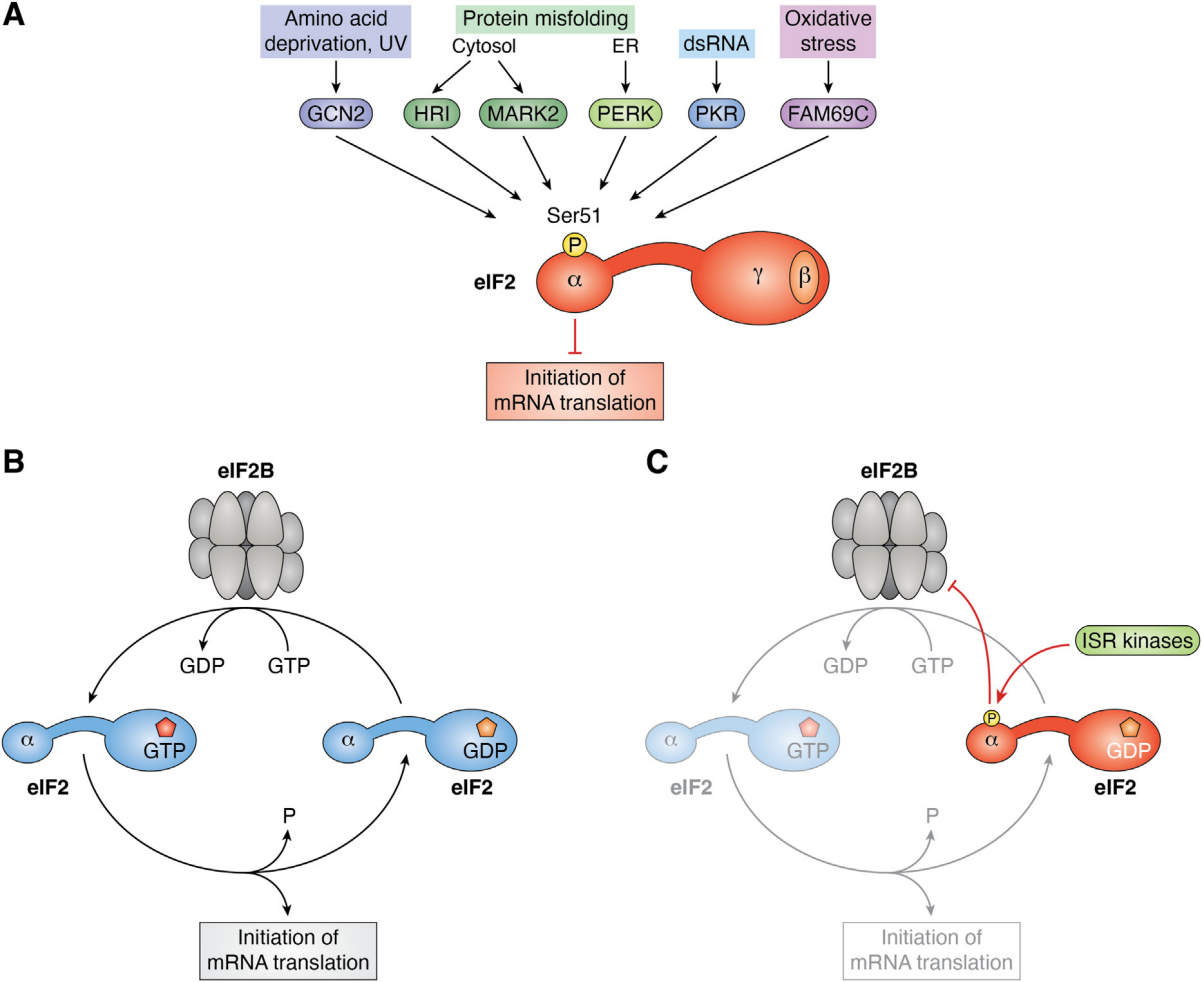
**The integrated stress response.***A*, kinases of the integrated stress response. Depicted are the kinases that become activated by the indicated types of cell stress. They all mediate phosphorylation of Ser51 of the α-subunit of eIF2, resulting in a blockade of mRNA translation initiation. *B* and *C*, regulation eIF2B by eIF2. *B*, recycling of eIF2•GDP. eIF2B exchanges the nucleotide on eIF2•GDP, thereby regenerating the active form eIF2•GTP that drives mRNA translation initiation. *C*, inhibition of eIF2•GDP recycling. ISR kinases phosphorylate the α subunit of eIF2, thereby turning it into an inhibitor of eIF2B and hence translation initiation. eIF2, eukaryotic translation initiation factor 2.

All these stress-activated kinases share a common substrate, the Ser51 residue of the α-subunit of eIF2, whose phosphorylation eventually leads to translation suppression (Fig. 1*A*). Hence, this mechanism has been termed the ISR (6). Despite the general protein synthesis shut-off, translation of some mRNAs, such as the one for transcription factor activating transcription factor 4, are even promoted under these circumstances, driving expression of genes that help to cope with the stress. This latter branch of the ISR is however not the subject of our review.

The core regulation step of the ISR is the blockade of eIF2B through ISR kinase-phosphorylated eIF2 [eIF2(αP)]. Normally, eIF2B acts by reloading GTP onto eIF2 in order to uphold the mRNA translation initiation cycle (Fig. 1*B*). eIF2B is a large and symmetric decamer with two specific sites at which GDP-loaded eIF2 is bound and GTP-loaded eIF2 released. Whereas unphosphorylated eIF2 is a substrate for eIF2B, eIF2(αP) is an inhibitor (Fig. 1*C*) that binds to a distinct site on eIF2B and induces a conformational switch that results in a loss of affinity for unphosphorylated eIF2.

The ISR is a fundamental cellular process common in eukaryotes, from yeast to human, to maintain protein homeostasis in cells and plays a key role in synaptic plasticity of higher animals. However, apoptosis is induced when the ISR is activated for a long period without an appropriate stress response or when the ISR is strongly activated by severe stress that cannot be resolved. This is a major problem in cells that are difficult to regenerate, such as neurons. In fact, it has been reported that the ISR is associated with several neurodegenerative diseases as well as other ailments (1, 7, 8, 9). Loss-of-function mutations in genes for eIF2B subunits can destabilize the complex, disturb the eIF2B-mediated reloading of GTP onto eIF2, or affect the binding of eIF2(αP), thereby causing translation dysregulations that can result in fatal neurological disorders or neonatal diabetes mellitus (1, 7, 8, 9).

## eIF2B and its regulation by eIF2

eIF2 consists of the three subunits α, β, and γ. The α and γ subunits harbor the phosphorylation and GTP/GDP-binding sites, respectively (1, 8, 10), and the β subunit binds to translation initiation factor eIF1, initiator methionyl-tRNA and eIF5 during the scanning process of translation initiation (11). To initiate translation, eIF2 transfers initiator methionyl-tRNA to the ribosome in a GTP-dependent manner. GTP is then hydrolyzed upon mRNA scanning and start codon recognition, and eIF2 is released from the ribosome. As the GDP-bound eIF2 is inactive, it needs to be returned to the active GTP-bound form by eIF2B, the eIF2-specific guanine nucleotide exchange factor (GEF). eIF2B consists of two sets of five different subunits (α to ε) (12, 13). The α_2_(βδ)_2_ hexameric regulatory subcomplex resides at the center, with two γε heterodimeric catalytic subcomplexes bound on opposite peripheral sides, together forming a decameric architecture with a 2-fold symmetric axis (14).

**Figure 2:**
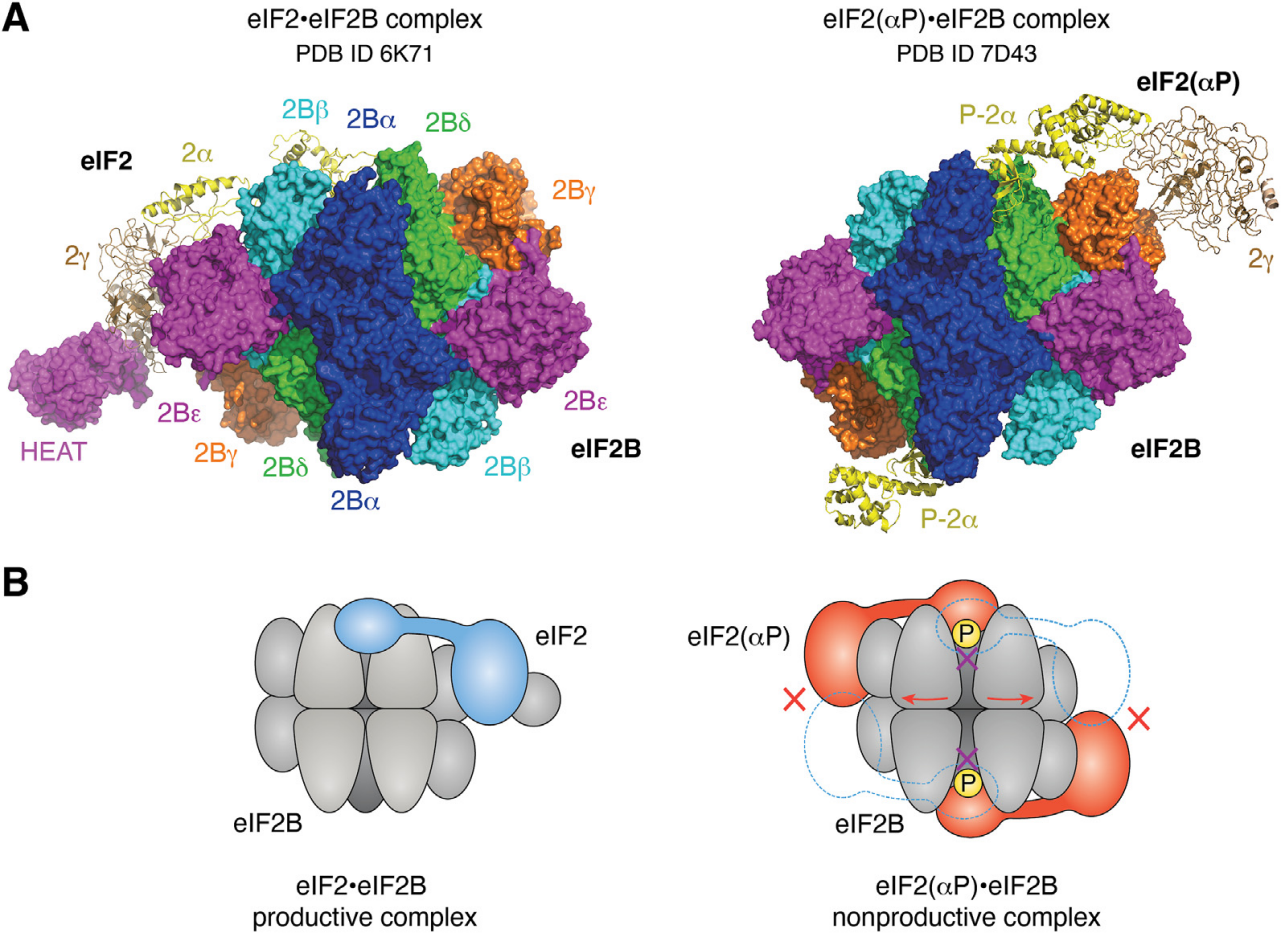
**The two types of complexes between eIF2B and eIF2.***A*, cryo-EM structures of eIF2•eIF2B (productive complex; PDB ID: 6K71) and eIF2(αP)•eIF2B (nonproductive complex; PDB ID: 7D43) (12, 13). eIF2B and eIF2 are represented with surface and cartoon models, respectively. *B*, schematic illustrations of the different binding sites of eIF2 and eIF2(αP) on eIF2B, resulting in formation of productive and nonproductive complexes, respectively. Please note that there are also structures showing that eIF2B is able to accommodate two molecules of eIF2 (PDB IDs 6O81 and 7F67). eIF2, eukaryotic translation initiation factor 2.

Despite their central importance for cellular physiology, the molecular details of both the eIF2B-mediated nucleotide exchange reaction on eIF2 and the inhibition of eIF2B by eIF2(αP) were long unresolved. Recently, these mechanisms were clarified by analyzing recombinantly expressed human eIF2B and eIF2 by means of cryo-EM (12, 13). Two types of complex structures were determined: the eIF2•eIF2B “productive” complex and the eIF2(αP)•eIF2B “nonproductive” complex. Importantly, it was found that eIF2 and eIF2(αP) bind to eIF2B in completely different regions (Fig. 2, *A* and *B*).

**Figure 3:**
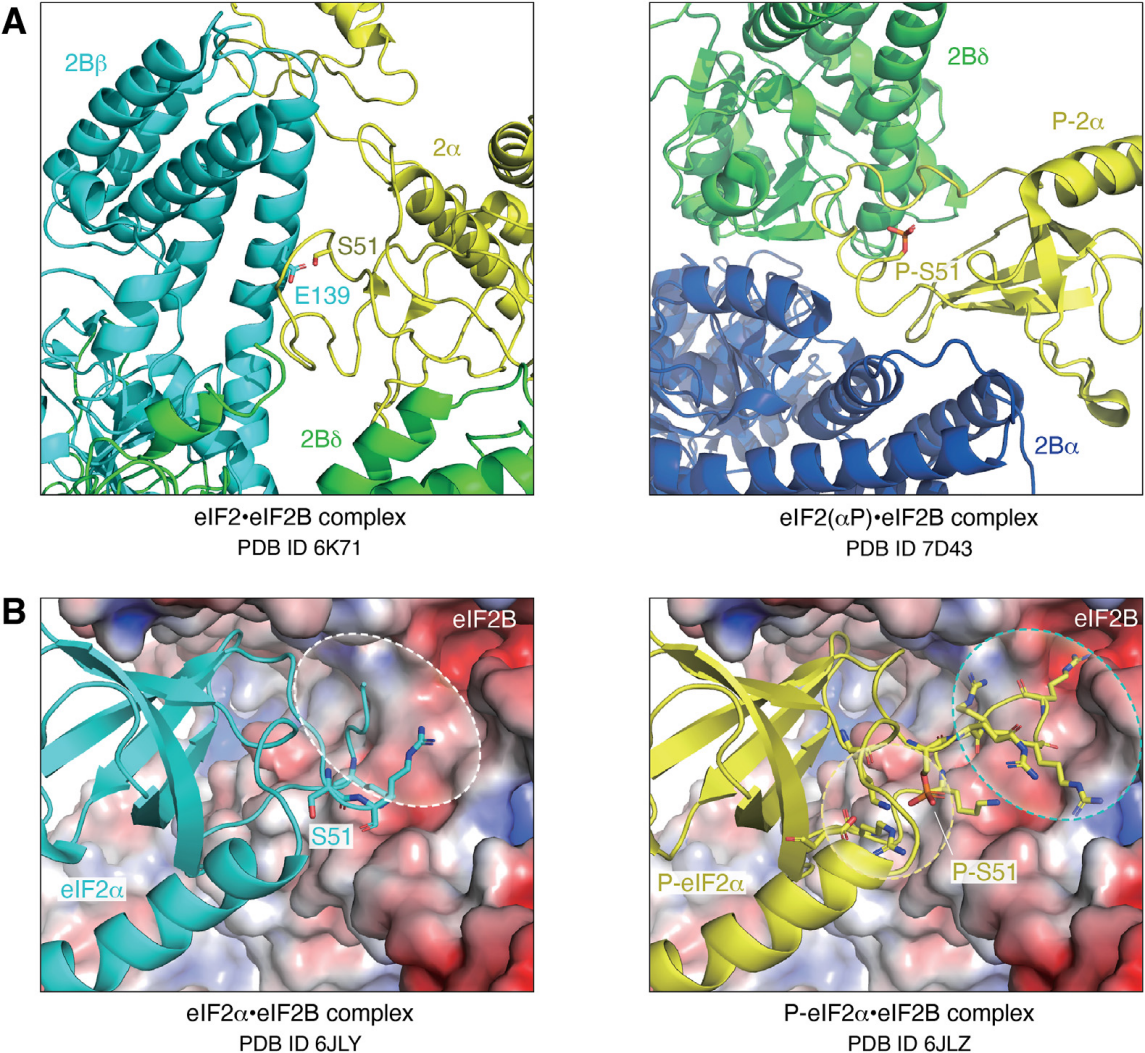
**eIF2•eIF2B and eIF2(αP)•eIF2B interactions.***A*, close-up of the interactions. Whereas unphosphorylated eIF2 is binding in a cavity between eIF2Bβ and eIF2Bδ (*left panel*), eIF2(αP) binds in a cavity between eIF2Bα and eIF2Bδ (*right panel*). *B*, inability of unphosphorylated eIF2 to occupy on eIF2B the binding site for phosphorylated eIF2. The crystal structures are of eIF2α•eIF2B (PDB ID: 6JLY) and P-eIF2α•eIF2B (PDB ID: 6JLZ). eIF2B is colored with electrostatic potential, where *red* and *blue colors* represent negative and positive potentials, respectively, of ±5.0 *kT*/*e*, generated by the program APBS (71). eIF2α alone can bind to the same pocket as P-eIF2α, but the loop following Ser51 does not form the stable structure and is disordered (*left panel*, *white dotted ellipse*). Thus, the binding is not stable. On the other hand, in the case of P-eIF2α, phosphorylated serine 51 forms the electrostatic network together with Arg63, Asp68, and Lys86 within eIF2α (*right panel*, *yellow dotted circle*), which leads to the conformational change of the Phos-S51-following loop (*right panel*, *blue dotted ellipse*). The loop contains many positively charged residues, such as Arg and Lys, which can stably bind to the negatively charged pocket of eIF2B (*colored red*). eIF2, eukaryotic translation initiation factor 2.

In the eIF2•eIF2B productive complex, the α-subunit of eIF2 (eIF2α) is recognized by the cavity between eIF2Bβ and eIF2Bδ. The side chain of Ser51 (the phosphorylation site) on eIF2α is recognized by the negatively charged side chain of Glu139 of eIF2Bβ (Fig. 3*A*, left panel, see also Fig. 2). As a side note, in the ISR the cavity between eIF2Bβ and eIF2Bδ is not likely to accept eIF2(αP) because the phosphorylated Ser51 (P-Ser51) of eIF2α and the Glu139 of eIF2Bβ would repel each other due to their negative charges. The γ subunit of eIF2 in the eIF2•eIF2B productive complex is sandwiched between the N-terminal body and the C-terminal catalytic HEAT (Huntington, Elongation Factor 3, PR65/A, TOR) domains of eIF2Bε. Recognition of the phosphate groups of GDP and GTP is mediated by the switch 1 region of the G domain of eIF2γ. Interestingly, in this complex, the switch 1 is widely open (different from a canonical structure), which is most likely the primary contributor to GDP dissociation. The majority of the β subunit of eIF2 is not present in the cryo-EM structure, as only one helix binding to the γ subunit was detectable. eIF2β is however known to bind to eIF2Bε to support the nucleotide exchange reaction (15).

In the eIF2(αP)•eIF2B nonproductive complex, phosphorylated eIF2α (P-eIF2α) is accommodated at another site, in the cavity between eIF2Bα and eIF2Bδ (Fig. 3*A*, right panel, see also Fig. 2). Intriguingly, P-Ser51 is not directly recognized, but rather plays a role in the structural reorganization of eIF2α that leads to a novel eIF2B–binding interface. This reorganization is achieved by P-Ser51 forming an intramolecular electrostatic network with other residues of eIF2α which in turn induces a conformational change in a positively charged loop on eIF2α C terminally to P-Ser51. In the absence of Ser51 phosphorylation, this loop region is disordered and not able to reach deep into the eIF2Bα/eIF2Bδ cavity (Fig. 3*B*, left panel) (12). However, with the P-Ser51-triggered electrostatic network the conformation-changed positively charged loop can deeply insert into the negatively charged pocket (Fig. 3*B*, right panel), which is likely responsible for the high affinity of eIF2(αP) to eIF2B. Importantly, when eIF2(αP) is bound in this nonproductive complex with eIF2B, the γ subunit of eIF2 no longer docks to eIF2Bε, but can to eIF2Bγ. Therefore, the catalytic HEAT domain of eIF2Bε is not located at a fixed position, and the complex is unable to promote the guanine nucleotide exchange reaction.

**Figure 4:**
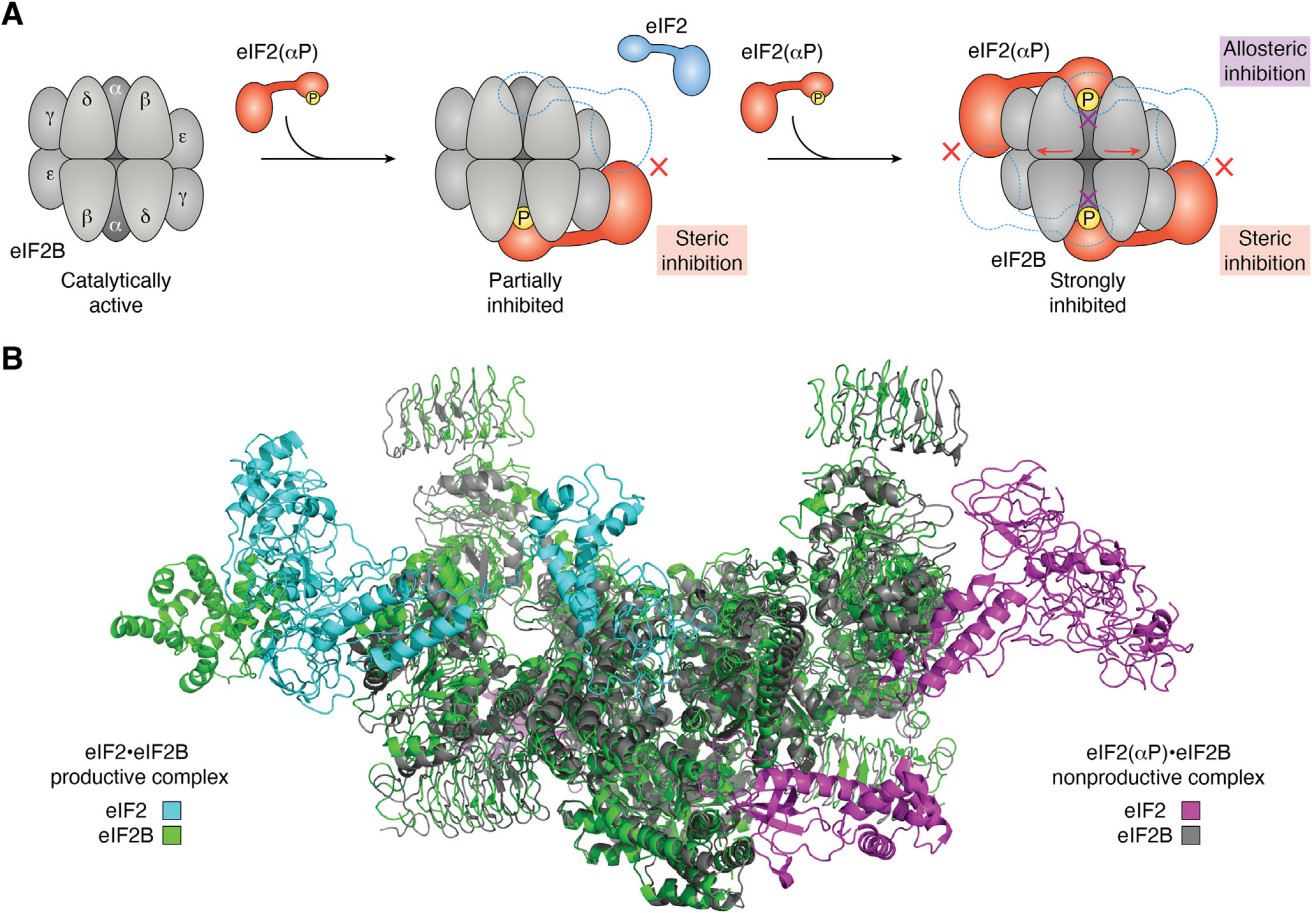
**Conformational states of eIF2B.***A*, *cartoons* are depicting the catalytically active state able to bind eIF2 and having GEF activity (*left*), the partially inhibited state imposed by eIF2(αP) to restrict the binding of eIF2 (*middle*), and the strongly inhibited state with a largely distorted eIF2-binding cavity. The two eIF2B subunits that become structurally perturbed upon eIF2(αP) binding are indicated by *red arrows*. Model is based on PDB structures 6K71 (eIF2B and eIF2) and 7D43 (eIF2B and P-eIF2α). *B*, structural difference of eIF2B between the eIF2-bound productive form (catalytically active, eIF2B: *green*, eIF2: *cyan*) and eIF2(αP)-bound nonproductive form (strongly inhibited, eIF2B: *gray*, eIF2 *magenta*). The eIF2Bα subunits, positioned at the *bottom*, are overlayed. It is visible that the eIF2α-binding surface of nonproductive eIF2B (*gray*) is shifted compared productive eIF2B (*green*). Note that even though the resolutions of 4.3 A each are not high, the movement of the subunits is big enough to detect. eIF2, eukaryotic translation initiation factor 2; GEF, guanine nucleotide exchange factor.

While it is clear how guanine nucleotide exchange on eIF2(αP) is inhibited, the question remains how eIF2(αP) prevents the nucleotide exchange activity of eIF2B for other, still unphosphorylated, eIF2 molecules. Through further structural studies of the eIF2•eIF2B and eIF2(αP)•eIF2B complexes, at least three different conformations of eIF2B have been identified: the catalytically active state, the partially inhibited state, and the strongly inhibited state (16, 17). The catalytically active state is apo eIF2B or eIF2B in the eIF2•eIF2B complex, which can accommodate eIF2 for the nucleotide exchange (Fig. 4*A*, left). The partially inhibited state is eIF2B bound by one molecule of eIF2(αP) (Fig. 4*A*, middle). In this state, the eIF2(αP) molecule bound on one side of eIF2B induces a slight structural change of eIF2B and sterically inhibits the eIF2 binding to the other side of eIF2B. The binding cavity for unphosphorylated eIF2α between eIF2Bβ and eIF2Bδ, however, remains similar to that observed in the fully active complex. The strongly inhibited state is eIF2B bound by two molecules of eIF2(αP) (Fig. 4*A*, right). In this state, the eIF2B structure is largely distorted and the eIF2-binding cavity is wider than in the other states, which results in allosteric inhibition of eIF2-binding to eIF2B. At the same time, the steric inhibition as in the partially inhibited state also functions for two eIF2(αP) molecules. Therefore, the strongly inhibited state imposed by two molecules of eIF2(αP) means that eIF2B is both sterically and allosterically inhibited for eIF2 binding. The structural perturbations of eIF2B when two eIF2(αP) molecules are bound are shown in Figure 4*B*. Besides these structural rearrangements, the Kd values for P-eIF2α and eIF2α binding to eIF2B were found to be 1.54 μM and 5.74 μM, respectively, indicating a preference for eIF2(αP) (12).

Table 1 lists PDB entries and details for all eIF2B complex structures that are currently available.

**Table 1:** Summary of available structures for eIF2B complexes

Sample	Species	Method	Resolution [Å]	PDB ID	Reference	Year
eIF2B apo	*Schizosaccharomyces pombe*	X-ray	3.0	5B04	(14)	2016
eIF2B apo	*Homo sapiens*	cryo-EM	2.8	7L70	(17)	2021
eIF2B apo	*H. sapiens*	cryo-EM	4.0	7D46	(16)	2021
eIF2B•eIF2α	*S. pombe*/*Saccharomyces cervisiae*	X-ray	3.5	6JLY	(12)	2019
eIF2B•P-eIF2α	*S. pombe*/*S. cervisiae*	X-ray	3.4	6JLZ	(12)	2019
eIF2B•P-eIF2α	*H. sapiens*	cryo-EM	3.0	6O9Z	(13)	2019
eIF2B•eIF2	*H. sapiens*	cryo-EM	4.3	6K71	(12)	2019
eIF2B•eIF2(αP)	*H. sapiens*	cryo-EM	4.3	6K72	(12)	2019
eIF2B•ISRIB	*H. sapiens*	cryo-EM	4.1	6EZO	(72)	2018
eIF2B•ISRIB	*H. sapiens*	cryo-EM	3.0	7L7G	(17)	2021
eIF2B•ISRIB•(eIF2)_2_	*H. sapiens*	cryo-EM	3.2	6O81	(13)	2019
eIF2B•ISRIB•eIF2	*H. sapiens*	cryo-EM	3.0	6O85	(13)	2019
eIF2B•F6P	*H. sapiens*	cryo-EM	2.9	7KMF	(73)	2021
eIF2B•eIF2(αP), α^P^γ comp.	*H. sapiens*	cryo-EM	4.3	7D43	(16)	2021
eIF2B•eIF2(αP), α^P^2 comp.	*H. sapiens*	cryo-EM	4.0	7D44	(16)	2021
eIF2B•eIF2(αP), α^P^1 comp.	*H. sapiens*	cryo-EM	3.8	7D45	(16)	2021
eIF2B•SFSV NSs	*H. sapiens*/SFSV	cryo-EM	2.3	7VLK	(55)	2021
eIF2B•SFSV NS	*H. sapiens*/SFSV	cryo-EM	2.6	7RLO	(56)	2021
eIF2B•SFSV NSs•eIF2	*H. sapiens*/SFSV	cryo-EM	2.8	7F66	(55)	2021
eIF2B•SFSV NSs•(eIF2)_2_	*H. sapiens*/SFSV	cryo-EM	3.6	7F67	(55)	2021
eIF2B(βH160D)	*H. sapiens*	cryo-EM	2.8	7TRJ	(74)	2022

## PKR

**Figure 5:**
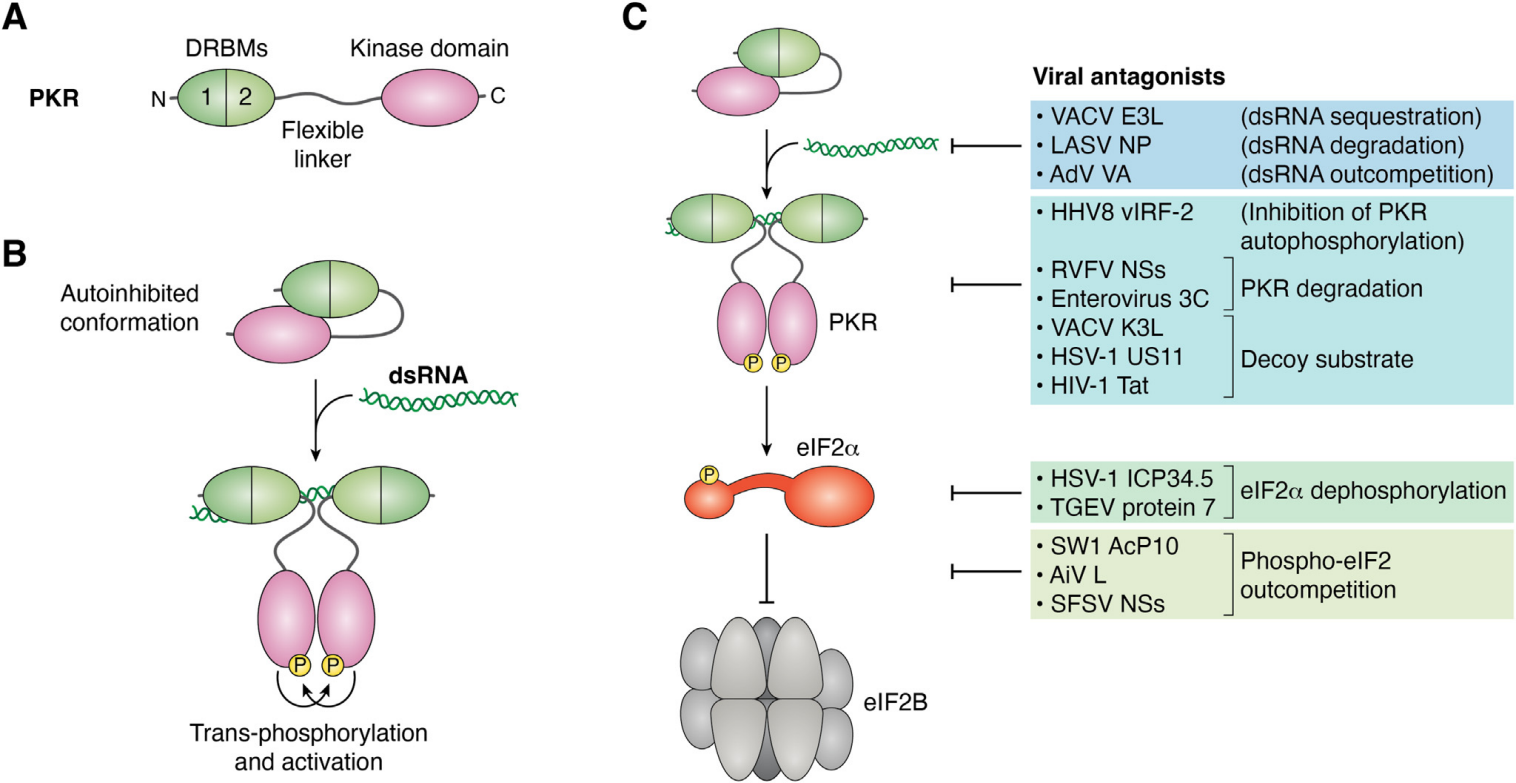
**Activation of PKR and viral antagonists.***A* and *B*, activation by dsRNA. *A*, arrangement of the PKR domains, with an N-terminal tandem DRBM, a flexible linker, and the C-terminal kinase domain. *B*, in the absence of the specific ligand dsRNA, the N terminus folds back onto the kinase domain, resulting in an autoinhibited closed conformation. dsRNA binding releases the DRBMs and the kinase domains from autoinhibition. Two cobinding PKR molecules transphosphorylate each other, resulting in activation. *C*, viral antagonists of the PKR pathway. Viruses have evolved manifold strategies to counteract the antiviral PKR pathway of eIF2B inhibition. Different viral factors interfere with different stages of the signaling chain (see also text). The three recently discovered antagonists shown on the bottom are directly interacting with eIF2B. DRBM, dsRNA-binding motif; eIF2, eukaryotic translation initiation factor 2; PKR, protein kinase RNA.

PKR, one of the four stress-activated serine/threonine kinases of the ISR, is specialized in antiviral defense (18, 19, 20). As a major player in innate immunity, PKR is constitutively expressed at low levels and upregulated by type I interferons (IFNs) (21). IFNs are cytokines that are produced when virus infections are sensed by cellular pattern recognition receptors, for example retinoic acid inducible gene I (RIG-I) (22, 23). Human PKR (also called eIF2 alpha kinase 2) has a length of 551 amino acids and contains an N-terminal domain with dsRNA-binding motifs (DRBMs) and a C-terminal kinase domain (Fig. 5*A*). The N- and C-terminal domains are separated from each other by a flexible linker. In the absence of dsRNA, the DRBMs bind to the kinase domain through an intramolecular manner, resulting in a closed conformation with an inactive kinase domain (Fig. 5*B*, top) (24). dsRNA is a by-product of viral replication (25) that can also be released from mitochondria under cellular stress (26). Upon binding of dsRNA (which does not need to be a perfect duplex (27)) to the DRBMs, the conformation opens up and the kinase domain is released (Fig. 5*B*, bottom). Moreover, when two PKR molecules bind to one stretch of dsRNA >30 bp, their kinase domains interact with each other, leading to a mutual “auto” phosphorylation of the monomers at Thr 446 and Thr 451 (27). The activated P-PKR is then able to interact with eIF2α *via* a specific α-helix in the C-terminal part of the kinase domain and phosphorylate it (22, 28). There is also a series of other substrates, for example IκB kinase β resulting in NF-kB activation (29) or p53 (30).

Besides these activities, PKR was also shown to be involved in inflammasome regulation (31), IFN induction by the pattern recognition receptor MDA5 (melanoma differentiation-associated gene 5) (32), stabilization of IFN mRNAs (33), induction of apoptosis (34), and the formation of stress granules *via* phosphorylation of eIF2α (35, 36).

Thus, PKR is a multifunctional antiviral effector of the IFN system. Its most potent mechanism, however, is the direct blockade of mRNA translation by the phosphorylation of eIF2α to convert it from a substrate to an inhibitor of eIF2B.

## Classical PKR antagonists of viruses

The central role of PKR in antiviral defense is highlighted by the plethora of countermeasures that viruses have evolved. Here, we will provide some examples that represent viral antagonists targeting the different steps of PKR activation and action (Fig. 5*C*). For more complete insights there are several excellent review articles (19, 24, 37, 38). At the start of the signaling chain, formation of PKR-activating viral dsRNA is either avoided by encapsidating complementary ssRNA by viral nucleoprotein (negative-sense RNA viruses (25)) or by hiding in membrane-enclosed compartments, a strategy employed by positive-strand RNA viruses including severe acute respiratory syndrome coronavirus 2 (SARS-CoV-2) (39). Some viruses also encode dsRNA-sequestering proteins (*e.g.*, E3L of vaccinia virus (40)) or immediately degrade the dsRNA by a specific exoribonuclease activity (Lassa virus nucleoprotein (41, 42)). An alternative strategy is to express either small dsRNAs that efficiently bind PKR but, due to particular structural features, block its subsequent activation (*e.g.*, virus-associated [VA] RNAs of adenoviruses (43)). There are also viral PKR-binding proteins that prevent PKR autophosphorylation (viral interferon regulatory factor-2 [vIRF-2] of human herpesvirus 8 (44)) or even act as a decoy substrate (*e.g.*, HIV-1 Tat, Herpes simplex virus-1 US11, vaccinia virus K3L (37)). A more radical solution to the same problem is exhibited by viruses destroying PKR, either by means of a virally encoded protease (enteroviral 3C (45)) or by tagging PKR for ubiquitination and degradation *via* the proteasomal system (Rift Valley fever virus NSs (46, 47)). While most viruses seem to intervene with the signaling chain at the levels of dsRNA or PKR, there are at least two viruses that act at the step of eIF2α. Human herpes simplex virus 1 as well as porcine transmissible gastroenteritis coronavirus encode proteins (ICP34.5 and accessory protein 7, respectively) that reverse the phosphorylation of eIF2α by recruiting its natural counterplayer, protein phosphatase 1α (48, 49).

## Novel PKR antagonists targeting eIF2B

The already quite heterogeneous group of viral PKR inhibitors recently got three new members that act even further downstream in the PKR-to-mRNA translation signaling chain (reviewed in (50, 51); see Fig. 5*C*, bottom). Beluga whale coronavirus (Bw-CoV) SW1, Aichi picornavirus (AiV), and Sandfly fever Sicilian phlebovirus (SFSV), despite being phylogenetically unrelated, all encode PKR antagonists that target the GEF eIF2B. Bw-CoV SW1 expresses the ORF 10-encoded accessory protein (AcP10) gene that was initially found to prevent the formation of stress granules and to rescue mRNA translation in the presence of eIF2(αP) (52). Subsequent analyses of AcP10 host cell interactors in human cells returned all three subunits of eIF2 as well as all five subunits of eIF2B. With respect to the eIF2 complex, there was a preference for the unphosphorylated eIF2α over phosphorylated eIF2α, indicating that AcP10 impedes the binding of phospho-eIF2α to eIF2B. In a similar vein, the gene product AiVL of the human pathogenic picornavirus AiV was able to rescue translation in the presence of eIF2(αP), to bind to eIF2B, and to reduce the binding of eIF2(αP) to eIF2B (52).

Bw-CoV and AiV are both positive-strand RNA viruses. By contrast the third virus, human pathogenic SFSV, is a phlebovirus with a mostly negative-stranded RNA genome. SFSV expresses a nonstructural protein, NSs, for which a large-scale comparative host protein interactome analysis had led to the discovery of the entire eIF2B complex as a host cell interactor (53). In line with this, SFSV NSs was found to enhance eIF2-dependent mRNA translation despite the presence of virus-activated P-PKR and eIF2(αP) (54). Moreover, SFSV NSs (and AiVL (52)) was only relevant for virus replication when host cells expressed a functional PKR, whereas in PKR-deficient cells it did not present a growth advantage. Thus, for both AiV and SFSV it is clear that PKR is the decisive ISR kinase, at least in cell culture. Therefore, PKR is the biological target of these viral antagonists even if they do not interact with it directly (similar to *e.g.*, ICP34.5 which dephosphorylates eIF2α (49)). Nonetheless, the targeting of eI2B for circumventing the PKR-mediated block of mRNA translation comes with the potentially beneficial side effect that the entire kinase arm of the ISR is paralyzed as well. Although there are currently no other cellular or viral examples besides SFSV, Bw-CoV, and AiV, the fact that three entirely different viruses are all targeting eIF2B may indicate that this anti-PKR strategy could be more widespread than appreciated.

## The productive state–protecting mechanism of SFSV NSs

**Figure 6:**
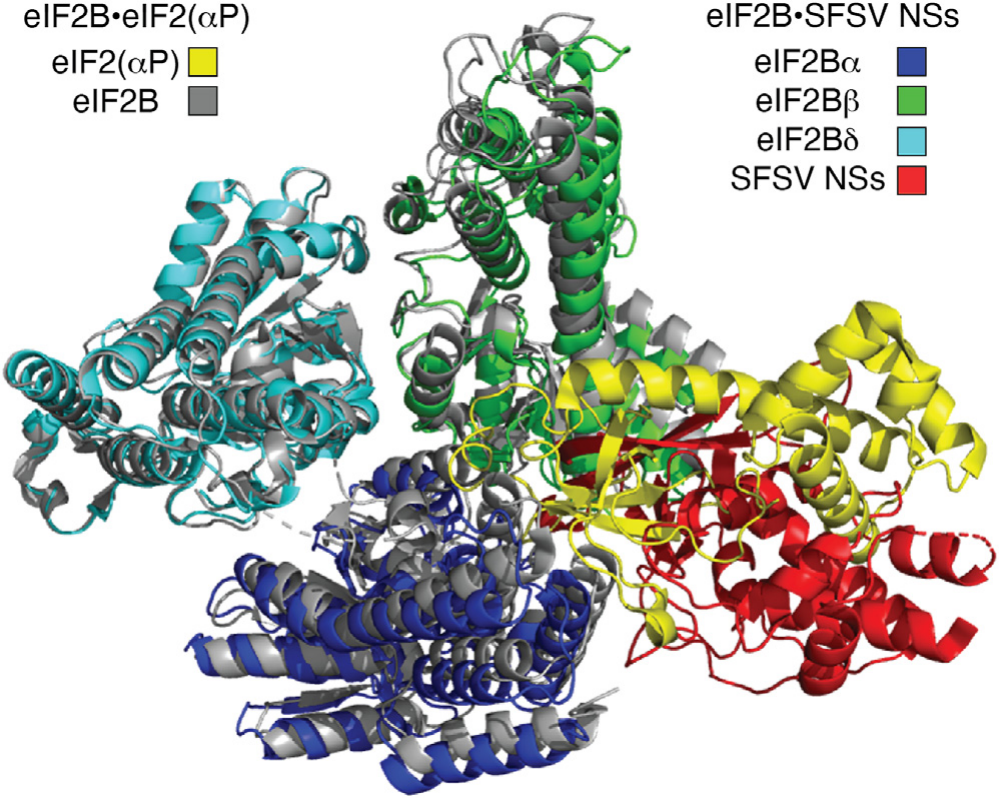
**Mechanism of SFSV NSs to protect eIF2B from inhibition by eIF2(αP).** Structure overlay of the eIF2B•eIF2(αP) complex (eIF2B: *gray*, eIF2(αP): *yellow*) and the eIF2B•SFSV NSs complex (eIF2Bα: *blue*, eIF2Bβ: *cyan*, eIF2Bδ: *green*, SFSV NSs: *red*), showing that eIF2(αP) and SFSV NSs are occupying the same “nonproductive cavity” on eIF2B. eIF2, eukaryotic translation initiation factor 2; NSs, nonstructural protein S segment; SFSV, Sandfly fever Sicilian phlebovirus.

**Figure 7:**
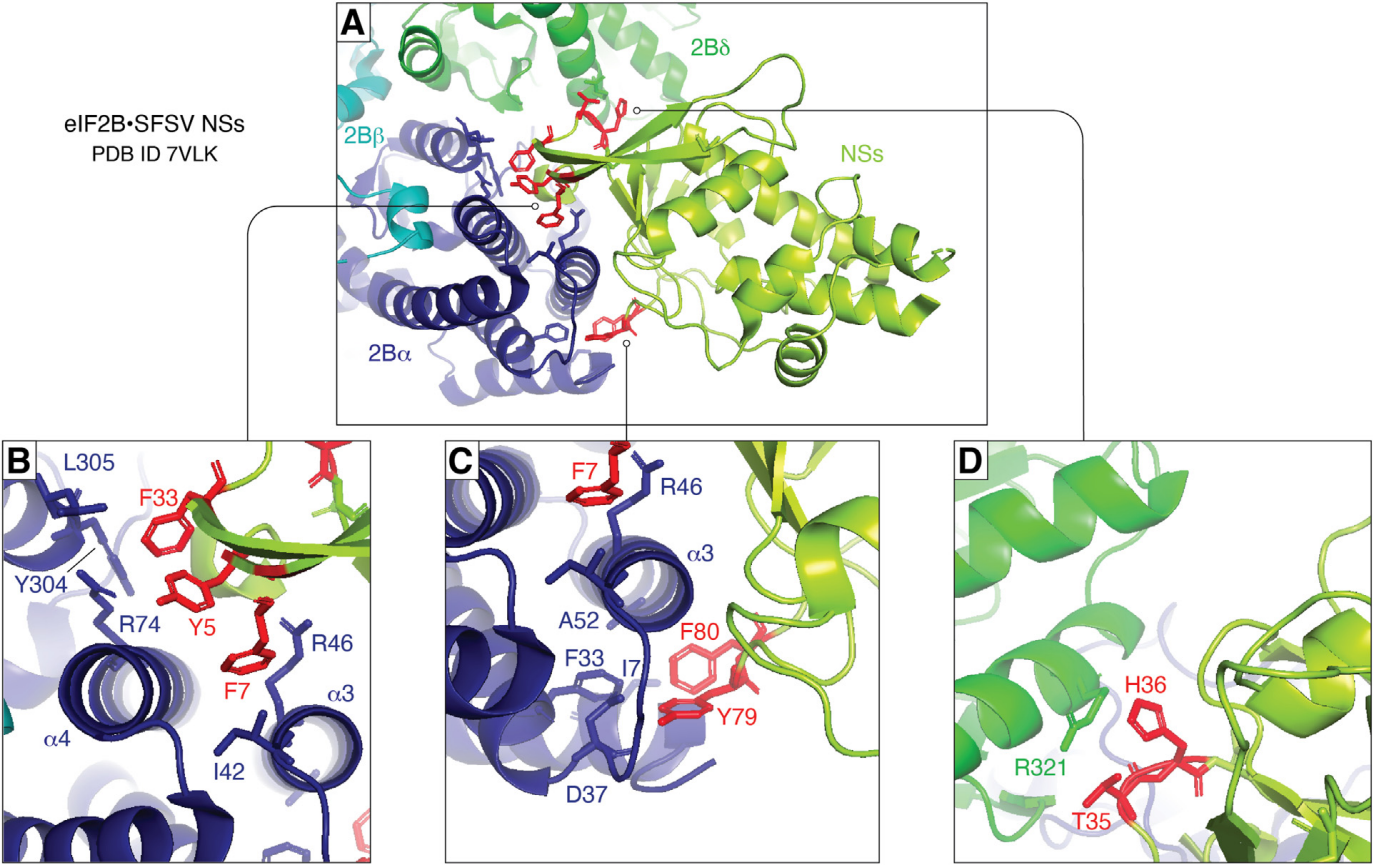
**Interactions of SFSV NSs with eIF2B.***A*, structure of the N terminus of NSs (*lime*) inserting into the cleft between eIF2Bα (*dark blue*) and eIF2Bδ (*green*) with the NSs residues that mediate eIF2B binding highlighted in *red* (PDB IDs: 7VLK [shown here] and 7RLO). *B*–*D*, detailed view of NSs–eIF2B interaction interfaces. *B*, NSs residues Y5, F7, and F33 make up aromatic cluster I, which contacts eIF2Bα helix α3 (predominantly *via* I42 and R46), helix α4 (*via* R74), and Y304 and L305. *C*, aromatic cluster II, comprising Y79 and F80, latches on to eIF2Bα helix α3 from the other side (involving A52) and additionally engages I7, F33, and D37. *D*, besides the aromatic clusters, NSs T35 and H36 contact eIF2Bδ R321. Note that residue D37 could not be shown because it points toward the opposite direction as eIF2Bδ R321 and NSs D37. eIF2, eukaryotic translation initiation factor 2; NSs, nonstructural protein S segment; SFSV, Sandfly fever Sicilian phlebovirus.

**Figure 8:**
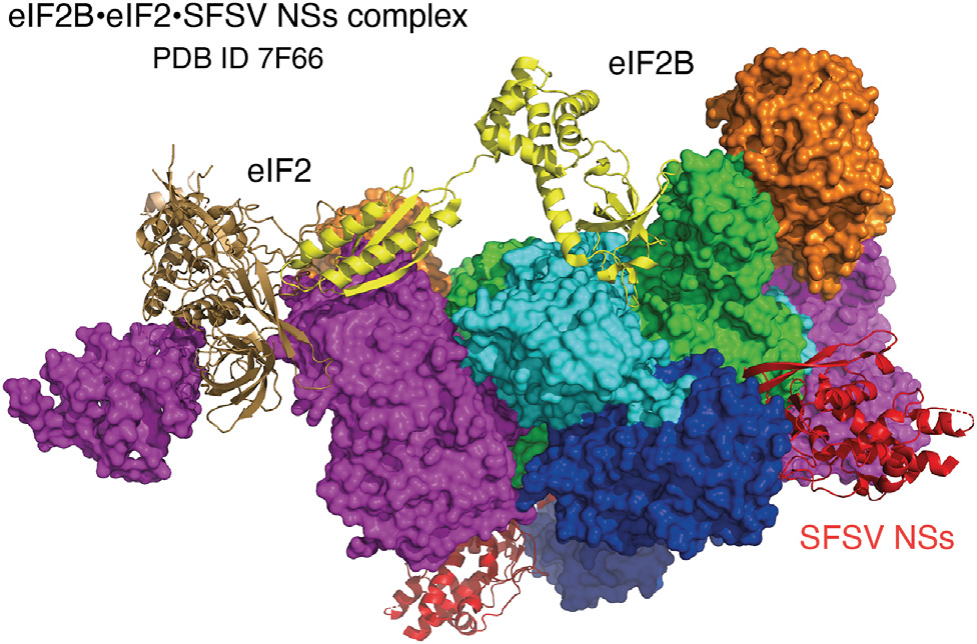
**eIF2B•eIF2•NSs complex.** Structure of the eIF2B•eIF2•SFSV NSs complex (PDB ID: 7F66). eIF2B and eIF2 are depicted as in Figure 3, and SFSV NSs is colored *red*. eIF2 and SFSV NSs can bind eIF2B simultaneously and there is no direct interaction between them. eIF2, eukaryotic translation initiation factor 2; NSs, nonstructural protein S segment; SFSV, Sandfly fever Sicilian phlebovirus.

Of the three novel viral antagonists that act at the level of eIF2B rather than further upstream, the atomic structure and molecular mechanism have been solved for SFSV NSs (55, 56). Cryo-EM structures showed that SFSV NSs binds in the same “nonproductive cavity” between eIF2Bα and eIF2Bδ as eIF2(αP) would occupy (Fig. 6). There are two aromatic clusters on the N terminus of NSs that interact with the α-helices of eIF2Bα by an extensive network of cation–π, hydrophobic, and polar interactions as well as hydrogen bonds (Fig. 7*A*). Aromatic cluster I consists of one tyrosine and two phenylalanines at positions 5, 7, and 33 (Fig. 7*B*), respectively, whereas aromatic cluster II consists of one tyrosine and one phenylalanine residues at positions 79 and 80, respectively (Fig. 7*C*). Besides these interactions with eIF2Bα, there is also binding to the eIF2Bδ subunit mediated by residues T35, H36, and D37 of SFSV NSs (Fig. 7*D*) (55, 56). Binding studies with NSs point mutants suggest that the interactions with eIF2Bδ are less important, whereas the aromatic clusters interacting with eIF2Bα are essential for the NSs–eIF2B interaction. As eIF2B is a decameric complex with two sets of each of its five subunits, it contains two binding sites for both unphosphorylated eIF2 and for eIF2(αP). Therefore, it can also accommodate two NSs molecules. However, although eIF2(αP) and NSs occupy the same cavity on eIF2B, eIF2(αP) broadly interacts with both the eIF2Bα and eIF2Bδ subunits, whereas NSs mainly interacts with eIF2Bα. The eIF2(αP) interaction with both eIF2Bα and eIF2Bδ induce structural changes, which widen and incapacitate the eIF2α-interacting pocket between the eIF2Bβ and eIF2Bδ subunits. Thus, only eIF2(αP) changes the conformation of eIF2B to inhibit the access of unphosphorylated eIF2 to the “productive” binding site (55, 56), the established mechanism by which eIF2(αP) inhibits mRNA translation (see above). NSs, by contrast, does not impose any major conformational changes, thus keeping eIF2B in the “productive” state that binds unphosphorylated eIF2 (Fig. 8). Moreover, due to its higher affinity, SFSV NSs outcompetes eIF2(αP) in entering eIF2B. As not all eIF2 molecules of an infected cell are phosphorylated at a given time (57) and levels of eIF2 are in general higher than of eIF2B (58), there are still substantial amounts of unphosphorylated eIF2 available to form the productive complex. Therefore, by obstructing the eIF2(αP)-binding site but preserving the productive conformation, SFSV NSs allows the binding of eIF2 to rescue the GEF activity of eIF2B even in the presence of high amounts of eIF2(αP). NSs is thus a stoichiometric protector of the productive eIF2B conformation. Consequently, SFSV NSs prevents not only translation inhibition by the antiviral eIF2α kinase PKR, but also the other ISR kinases PERK, GCN2, and HRI (55, 56). However, in the viral context a lack of NSs (or AiVL) could be compensated for by knockdown or knockout of PKR (52, 54). Although viruses are known to also cause ER stress (59), this suggests that inhibiting the PKR pathway is the main purpose of the viral eIF2B protectors.

It is also of note that, although SFSV NSs and eIF2(αP) bind in the same cavity on eIF2B, there is no similarity in either sequence or structure (51, 55, 56). This indicates that stoichiometric protection of eIF2B from eIF2(αP) enjoys a certain degree of freedom and suggests that there may be other such inhibitors encoded by viruses or perhaps even by cells. In line with this, the phlebovirus NSs proteins were suggested to share a similar core domain fold (60), whereas the N and C termini are known to harbor host protein-binding sites, indicating genetic flexibility (47, 55, 56, 61).

## Clinical application of ISR inhibitors

The ISR is associated with many genetic and age-related diseases with metabolic or cognitive disorders, macular degeneration, as well as with cancer (1, 7, 8, 9, 62). Specific mutations in eIF2B subunits can disturb normal physiology and development, leading to neonatal diabetes, liver dysfunction, or to a fatal neurodegeneration (vanishing white matter disease). Diabetes and liver dysfunction can also be caused by eIF2 mutations. Cancer cells often overexpress eIF2B in order to maintain protein synthesis at high level (63). Mutations in PERK cause the rare Wolcott–Rallison syndrome that again involves, for example, diabetes and neurodegeneration. Moreover, the activity of the ISR increases with aging (64), and increased ISR activity was reported for neurodegenerative diseases like Alzheimer’s, Parkinson’s, or amyotrophic lateral sclerosis, to name a few (7).

In 2013, the small molecule integrated stress response inhibitor (ISRIB) was reported to counteract the detrimental effects of eIF2(αP) (65). Interestingly, it has been revealed that ISRIB binds to the pocket between eIF2Bβ and eIF2Bδ on the 2-fold symmetry axis of eIF2B and functions as an allosteric inhibitor for the interaction between eIF2B and eIF2(αP) (16, 17). Thus, the compound acts similar to SFSV NSs but through an entirely different mechanism. ISRIB was reported to work effectively in several neurodegenerative disease model organisms (7), such as prion disease (66), traumatic brain injury (67, 68), and Alzheimer’s disease (69). In line with this, we recently showed that SFSV NSs could also protect cultured neurons from mRNA translation repression by ER stress (55). ISRIB also improved learning in some but not all mouse models (65, 70). Therefore, treatments that control the ISR may hold great promise with respect to a wide array of rare as well as common human diseases.

## Conclusions and outlook

Although the ISR is an important and well-established phenomenon at the center of both cellular and physical integrity, new aspects are constantly coming to the light. Now that the atomic structure of eIF2B in the productive and nonproductive states are solved, it will become easier to design small molecules that specifically interfere with an overactive or chronic ISR. It is hoped that the sophisticated mechanism by which SFSV NSs shields the eIF2(αP)-binding cavity, while maintaining the productive eIF2B mode may help to further accelerate the developments in this exciting field of basic and applied research.

## Conflict of interest

The authors declare that they have no conflicts of interest with the contents of this article.
